# Simultaneous detection of eight cancer types using a multiplex droplet digital PCR assay

**DOI:** 10.1002/1878-0261.13708

**Published:** 2024-09-06

**Authors:** Isabelle Neefs, Nele De Meulenaere, Thomas Vanpoucke, Janah Vandenhoeck, Dieter Peeters, Marc Peeters, Guy Van Camp, Ken Op de Beeck

**Affiliations:** ^1^ Center of Medical Genetics University of Antwerp and Antwerp University Hospital Edegem Belgium; ^2^ Center for Oncological Research University of Antwerp and Antwerp University Hospital Wilrijk Belgium; ^3^ Department of Pathology Antwerp University Hospital Edegem Belgium

**Keywords:** biomarkers, ddPCR, DNA methylation, multi‐cancer detection, multiplexing

## Abstract

DNA methylation biomarkers have emerged as promising tools for cancer detection. Common methylation patterns across tumor types allow multi‐cancer detection. Droplet digital PCR (ddPCR) has gained considerable attention for methylation detection. However, multi‐cancer detection using multiple targets in ddPCR has never been performed before. Therefore, we developed a multiplex ddPCR assay for multi‐cancer detection. Based on previous data analyses using The Cancer Genome Atlas (TCGA), we selected differentially methylated targets for eight frequent tumor types (lung, breast, colorectal, prostate, pancreatic, head and neck, liver, and esophageal cancer). Three targets were validated using ddPCR in 103 tumor and 109 normal adjacent fresh frozen samples. Two distinct ddPCR assays were successfully developed. Output data from both assays is combined to obtain a read‐out from the three targets together. Our overall ddPCR assay has a cross‐validated area under the curve (cvAUC) of 0.948. Performance between distinct cancer types varies, with sensitivities ranging from 53.8% to 100% and specificities ranging from 80% to 100%. Compared to previously published single‐target parameters, we show that combining targets can drastically increase sensitivity and specificity, while lowering DNA input. In conclusion, we are the first to report a multi‐cancer methylation ddPCR assay, which allows for highly accurate tumor predictions.

## Graphical abstract

DNA methylation biomarkers show promise for cancer detection. Multi‐cancer detection via common methylation patterns is possible, for which droplet digital PCR (ddPCR) is a key tool. We developed a multiplex ddPCR assay using three differentially methylated targets for simultaneous detection of eight tumor types. With an accuracy of 95%, our assay drastically improves sensitivity and specificity compared to single‐target methods.

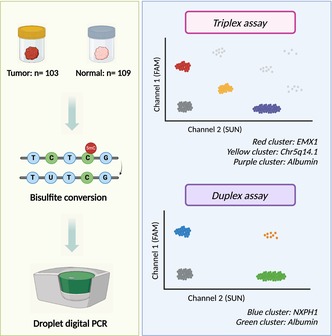

**Abbreviations**
%CV: intra‐class coefficient of variability
cfDNA: cell‐free DNA
cvAUC: cross‐validated area under the curve
ddPCR: droplet digital PCR
*EMX1*
: empty spiracles‐like protein 1
HCC: hepatocellular carcinoma
LOB: limit of blank
LOD: limit of detection
NFQ‐MGB: non‐fluorescent quencher minor groove binder
*NXPH1*
: neurexophilin 1
ROC: receiver‐operator characteristic
TCGA: The Cancer Genome Atlas
TcP: tumor cell percentage
TOO: tissue of origin

## 1 Introduction

Cancer is one of the leading causes of death worldwide, with nearly 10 million deaths reported in 2020. The most common cancers are breast (2.26 million new cases), lung (2.21 million new cases), colorectal (1.93 million new cases) and prostate (1.41 million new cases) [1]. Many cancers can be cured when treated efficiently and, more importantly, detected early. However, patients are still frequently diagnosed in a late stage [2], despite efforts in screening programs and early detection methods. For example, 68% of lung cancer and 59% of colorectal cancer patients is diagnosed in stage IV [2], where 5‐year survival rates are 7% and 14%, respectively. There are currently only screening programs in place for colorectal, breast, and cervical cancer in most western countries, and in some countries (e.g. the United States), screening for lung cancer is also recommended [3]. It is clear that there is much room to improve on cancer detection, especially in early stages. Therefore, early detection biomarkers have gained interest in recent years. DNA methylation is a very good candidate for such biomarkers. It occurs at the 5th position of cytosines in the context of CpG dinucleotides. Tumor‐associated modifications of the methylation status of CpG sites appear already early in carcinogenesis, possibly even before actual neoplastic transformation [4, 5]. This makes DNA methylation changes the ideal target for early cancer detection. Methylation patterns are extensively altered between normal cells and cancer cells and are a very consistent feature as opposed to mutations, which typically vary at a wide range of sites [6, 7]. Therefore, DNA methylation assays can be used off‐the shelf, making them faster and cheaper to use compared to other assays. Distinct methylation patterns per tumor type have been observed and allow detection of tissue of origin. In addition,common methylation patterns exist across tumor types, which allow multi‐cancer detection [7, 8]. This multi‐cancer detection has gained more attention in the past few years. Different assays are being developed and tested in clinical trials for safety and effectiveness [9, 10]. The advantage of such multi‐cancer detection tests is that they could facilitate the early detection of many cancers for which currently, no site‐specific screening modality exists. Moreover, they could also detect cancers that are missed by the existing screening tests, as these assays are more sensitive than for example the existing imaging tests [10, 11].

Just a decade ago, droplet digital PCR (ddPCR) was introduced as a highly sensitive method for detection and absolute quantification of targeted mutations in DNA [12, 13]. The random partitioning of a clinical sample into thousands of droplets allows simultaneous PCR reactions in each individual droplet, drastically improving sensitivity [12, 14, 15]. In 2015, ddPCR was used for the first time to detect methylated alleles [16]. Since then, several studies have explored DNA methylation biomarkers in ddPCR assays for cancer detection and follow‐up, mostly in liquid biopsies [6, 7, 13, 17, 18, 19, 20]. Up until this moment, there are no methylation ddPCR assays FDA‐approved for use in clinical settings. None of the methylation ddPCR assays that are published, focused on multi‐cancer detection using multiple (candidate) biomarkers. The multi‐cancer tests that have been developed thus far, for example the Galleri® or PanSeer test usually make use of next‐generation sequencing technologies [21, 22, 23], although these technologies are inferior to ddPCR regarding detection sensitivity, more time‐consuming and costly [13, 18].

Previous research from our group has demonstrated the possibility of using DNA methylation biomarkers to discriminate eight different tumor tissues from each other and from normal tissue. The analyses were performed *in silico*, and our validated prediction model with 20 CpGs, common for the different cancer types, achieved 85% sensitivity at 91% specificity for stage one cancers [8]. However, efforts to design multiplex methylation assays often result in high‐cost tests that are not affordable for routine diagnostics. Therefore, we aimed to develop and validate a triplex and duplex ddPCR assay for the detection of eight cancer types in fresh frozen tissue based on three differentially methylated targets.

## 2 Materials and methods

**Fig. 1:**
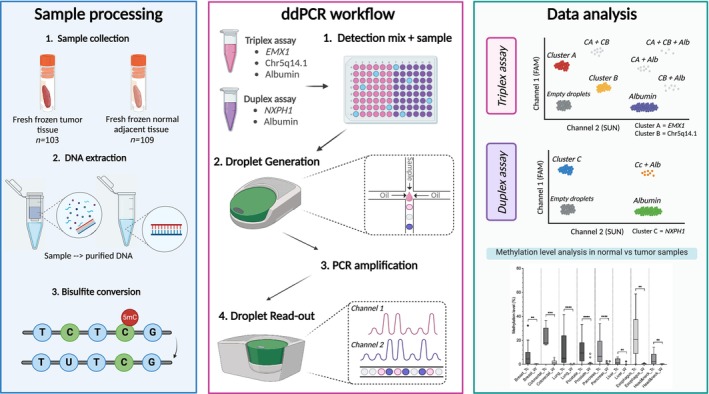
Overview of the materials and methods of the study. In the data analysis, clusters in gray indicate the double (or triple) positive clusters. Droplets of these clusters are excluded from the data. ddPCR, droplet digital PCR.

An overview of the methods is given in Fig. 1.

### 2.1 Sample collection and characteristics

#### 2.1.1 Control samples

Whole blood samples (*n* = 20) were previously collected from healthy volunteers at the University Hospital of Antwerp in January 2020. They were used as negative controls and for determination of the limit of blank (LOB) and limit of detection (LOD) (see Section 2.5 for explanation). In addition, the HCT116 (CRC; RRID: CVCL_0291) and Cal27 (Head and neck; RRID: CVCL_1107) cancer cell lines were used as positive controls. Cell lines were provided by the Centre for Oncological Research (CORE). They were cultured according to the standard protocols from the American Type Culture Collection (ATCC, Manassas, VA, USA). The cell lines were authenticated at the start of the study using short tandem repeat profiling with the PowerPlex® 16 system (Promega, Madison, WI, USA). They were routinely tested for mycoplasma contamination, which was negative.

#### 2.1.2 Fresh frozen tissue

Tumor tissue samples and normal adjacent tissue samples for eight cancer types were retrospectively obtained from the Antwerp University Hospital biobank (UZA, Belgium). Samples were collected from February 2012 to January 2023. In total, 103 tumor and 109 normal adjacent tissue samples (Table 1) were collected and stored at −80 °C. For all specimens, a hematoxylin and eosin‐stained slide was made for microscopic analysis by the pathologist. Tissue type, presence of invasive tumor and overall tumor cell percentage (TcP) were verified. The average TcP was 40%, ranging from 5% to 95%. Samples from all invasive cancer stages (I–IV) were included in the study. The sample size calculation was performed as described in [24]. More details are given in the Statistics section 2.6.

**Table 1:** Tissue type and sample numbers used for ddPCR assays.

Tissue type	Histological subtype	No. of tumor	No. of normal adjacent
Lung	Adenocarcinoma and squamous cell carcinoma	20	22
Breast	Invasive carcinoma	10	7
Colorectal	Adenocarcinoma	7	10
Pancreas	Adenocarcinoma	17	23
Liver	Hepatocellular carcinoma	12	10
Esophagus	Squamous cell carcinoma	10	5
Head and neck	Squamous cell carcinoma	13	8
Prostate	Adenocarcinoma	14	24
Total		103	109

### 2.2 DNA extraction and bisulfite conversion

For whole blood samples, genomic DNA (gDNA) was extracted from 50 mL blood using a standard salting‐out process. The DNA was eluted in 1.5 mL TE buffer and stored at 4 °C until further use. Genomic DNA from the cell lines was extracted from 10^6^ cells using the Blood & Cell Culture DNA Maxi Kit (Qiagen, Hilden, Germany). DNA was eluted in 500 μL TE buffer and was stored at −20 °C until further use. From fresh frozen tissue, genomic DNA was extracted from 1 to 10 mg tissue using the QIAamp DNA Micro kit (Qiagen). DNA was eluted in 70 μL TE buffer and stored at −20 °C until further use. DNA concentrations were measured using the Qubit Fluorometer 2.0, DNA broad range assay (Invitrogen, Waltham, MA, USA). Bisulfite conversion (20 ng per sample and per assay) was performed using the EZ DNA Methylation kit (Zymo Research, Irvine, CA, USA) according to the manufacturer's instructions. The elution volume was set to 2 μL × *n* + 1 μL, where *n* was the number of assays (usually 2), and 1 μL was added as a margin for technical errors. Bisulfite converted DNA was stored at −20 °C and used within 10 days after conversion. We used 20 ng to have sufficient material for optimization and validation of the assays, in view of addressing potential issues e.g. rain in the assay.

### 2.3 Assay development

#### 2.3.1 Target selection process

Targets were discovered through statistical analysis (pairwise comparison of cancer vs normal adjacent samples) of large datasets from The Cancer Genome Atlas. In total, 1792 differentially methylated CpG sites were found. A total of 40 targets with a *P*‐value of < 0.01 and an absolute difference of 20% in tumor vs control methylation level were selected for primer design. This selection was based on (a) targets with the lowest *P*‐value, (b) targets with the largest difference in methylation level and (c) targets for which primers could be designed. Of these 40 targets, the 27 best performing primer sets *in silico* were ordered from Integrated DNA technologies (IDT, Leuven, Belgium). Primers were evaluated using qPCR (384 CFX; Bio‐Rad, Hercules, CA, USA) on bisulfite converted gDNA from whole blood samples and cell lines. The targets with the largest differences in *C*_q_ values and the best matching melting temperatures were selected, which ultimately led to the retention of three targets for ddPCR assay development. Compared to the previous study [8], different targets were used due to technical reasons. For two of the probes that were previously described, primers could not be designed. For one of the probes, a SNP was present. For the last probe, the *in silico* primer conditions (melting temperature, %CG content, self‐ligation…) were less optimal.

#### 2.3.2 Primer and probe design for targets

Primers for target 1 were designed using *in silico* bisulfite conversion through the serial cloner software (version 2.6.1) and primer3plus (bioinformatics.nl/cgi‐bin/primer3plus/primer3plus.cgi). Primers for targets 2 and 3 were designed using meth primer (urogene.org/cgibin/meth‐primer/methprimer.cgi). Primers were ordered from Integrated DNA technologies (IDT, Leuven, Belgium). Probes were manually designed based on *in silico* bisulfite converted sequences. Non‐fluorescent quencher, minor groove binder (NFQ‐MGB) probes were used (IDT, Coralville, IA, USA). An overview of the newly designed primer and probe sequences can be found in Table 2. For albumin, primers were previously designed by Boeckx et al. [20]. The fluorophore from the published albumin probe was adapted from FAM to SUN, an equivalent for the VIC dye.

**Table 2:** Newly developed primer and probe sequences for the triplex ddPCR assays. N*, NFQ‐MGB = non‐fluorescent quencher – minor groove binder.

Target	1	2	3
Target name	*EMX1*	Chr5q14.1	*NXPH1*
Amplicon location	2:73147755–73147844+	5:76923876–76923965+	7:8482030–8482119+
Amplicon length	100 bp	87 bp	82 bp
Forward primer	5′CGAACGAAAAGGAATATGTTTG3′	5′GATACGTTTTTTTTGGAGAAGCGC3′	5′GAAGCGAAGGATTTTAGTTGTCG3′
Reverse primer	5′CTTCCAACGCCTCGATTAAC3′	5′CTTCATATCCCCAAACCCGAA3′	5′GAATACCCTCTCCTTCCGATATAACGA3′
Probe(s)	FAM‐CGGCGCGGTTTCGGCG‐N*	FAM‐TGGGAGGTTTCGGGTATTTGAAGCG‐N*	FAM‐CGTAGGGGGAGGTCGCGCG‐N*
		SUN‐TGGGAGGTTTCGGGTATTTGAAGCG‐N*	

#### 2.3.3 Optimization of the assays (temperature and concentration)

The assays were first assessed using a temperature gradient between 52 °C and 62 °C. The optimal temperature was chosen to further optimize the probe concentrations. Primer concentrations were adopted from Boeckx et al. [20]. In the triplex assay, target 1 was detected based on 6‐FAM fluorescence and target two was detected based on both 6‐FAM and 6‐SUN fluorescence. By combining the two different fluorophores on the same probe, a third color was artificially created for detection. By adjusting (increasing/decreasing) the ratio between 6‐FAM and 6‐SUN for the target that is detected in both channels, the clusters could be separated more (see also Fig. S1). In the duplex assay, target 3 was detected based on 6‐FAM fluorescence. In both assays, albumin was used as a reference to determine the total amount of DNA present. Albumin detection was based on 6‐SUN fluorescence, for which the probe concentration was adopted from Boeckx et al. [20]. After complete concentration optimization, a final temperature gradient around the previously chosen temperature was used to determine the optimal temperature for both assays.

### 2.4 ddPCR workflow

#### 2.4.1 Assay composition

First, the assay mix containing primers, probes, and Tris–HCl (pH 8) was made. Assay mixes were optimized as described above. Subsequently, the detection mix was composed of 11 μL 2× Bio‐Rad ddPCR™ Supermix for probes (no dUTP), 1.1 μL of the assay mix, 2 μL DNA and 7.9 μL Milli‐Q H_2_O to a final volume of 22 μL. For both assays, the target and reference primer concentrations as well as the reference probe concentration were 900 nm in the final detection mix. Optimized concentrations for the target probes are given in the results section. Approximately 20 000 nL sized droplets were generated according to the manufacturer's protocol using the automated QX200™ Droplet generator (Bio‐Rad). The resulting droplets were transferred to a 96‐well plate and sealed. Target sequences were amplified using a Veriti™ thermal cycler (Applied Biosystems, Waltham, MA, USA). Temperatures and times for the activation and inactivation steps were based on the recommended protocol from Bio‐Rad for the ddPCR™ Supermix for Probes (no dUTP) (see Table S1). Ramp rates of 2.5 °C·s^−1^ were used based on Bio‐Rad guidelines. The amplification temperature was set at the optimized temperature, given in the results section. An additional incubation step at 12 °C for (at least) 15 min was added to the recommended Bio‐Rad cycling protocol. Samples were immediately analyzed post amplification in the QX200™ Droplet Reader (Bio‐Rad).

#### 2.4.2 Sample analysis

**Fig. 2:**
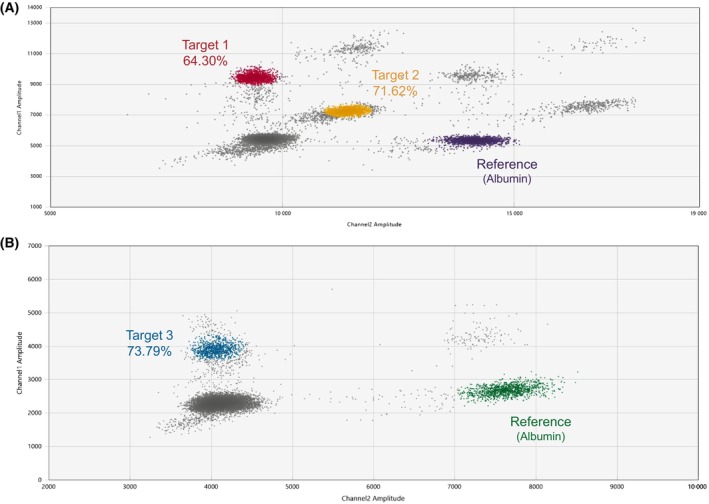
Dispersion graphs of the triplex and duplex assay. The graphs show the dispersion of droplets for a positive control (HCT116, CRC cell line). (A) Triplex assay consisting of target 1, 2 and reference Albumin. (B) Duplex assay consisting of target 3 and reference albumin. Both graphs show an example of one replicate experiment, but are representative for all experiments (*n* = 15 for optimization and characteristic measurements and *n* = 10 for fresh frozen runs) including the positive control.

After read‐out, the data was analyzed using the quantasoft™ analysis pro software (version 1.0.596; Bio‐Rad). This software was used to visualize the dispersion graphs. The positive control was used to set clusters based on fluorescence detection in the 6‐FAM and/or 6‐SUN channel. Four different clusters were manually assigned for the triplex assay (see Fig. 2A). The different clusters are (a) empty droplets (shown in gray in Fig. 2), (b) droplets containing the reference sequence albumin (shown in purple in Fig. 2A), (c) droplets with the first target sequence (shown in red in Fig. 2A) and (d) droplets containing the second target sequence (shown in yellow on Fig. 2A). Similarly, three distinct clusters for the duplex assay were manually assigned (Fig. 2B). Here, the reference sequence is given in green (Fig. 2B) and target 3 is shown as blue droplets (Fig. 2B). Droplets from double and triple positive clusters are excluded from all analyses [25]. A cut‐off of at least 10 000 accepted droplets and at least 1000 droplets for albumin (~ 4 ng as the lower limit of DNA input we accept) was set for analyzing all ddPCR experiments. The range of DNA varied from 4 to 19.72 ng, with an average of 9.47 ng. For samples in which the number of droplets was lower, the ddPCR protocol was repeated. Examples of a negative sample for both the triplex and the duplex assay is given in Fig. S1.

### 2.5 Assay characteristic measures

The limit of blank (LOB) is described as the number of false positive droplets that is detected in the negative controls (human genomic DNA) [26]. The LOB was used to discriminate the positive droplets (i.e. droplets containing the methylated target) from noise by subtraction of the LOB from detected positive droplets. The LOB was calculated as described in [20] and in Table S2. For both assays, the LOB was defined by the number of positive droplets measured in gDNA from whole blood samples that are not hypermethylated (*n* = 19 for the triplex assay, *n* = 20 for the duplex assay). All data given in the results is normalized and corrected with the LOB (see also Section 2.5.1). The limit of detection (LOD) is defined as ‘the minimum concentration of the rare sequence that can be reliably differentiated from a negative control’ (see also Table S2) [26]. To measure this minimal detectable methylation level, a DNA titration experiment using human methylated and non‐methylated (WGA) DNA (Zymo Research) was performed. Five replicates of concentrations between 0% and 5% were measured with the assays to determine the LODs. They were calculated as described in [27] (see also Table S2). To determine the corresponding methylation level, linear regression was used. The minimally detectable levels, thus LODs, and are given in Table 3. The intra‐assay coefficient of variability (%CV) demonstrates the variance between sample replicates within the assays and is a measure of repeatability. The copies per microliter of four positive controls was used to calculate the %CV as described in Table S2. The LOB, LOD and %CV of the targets is given in Table 3. The analytical detection sensitivity is a measure of the number of DNA copies that can be reliably detected. It was calculated by dividing the number of haploid genome equivalents (3) by the number of copies detected per well (in percentage, Table S2) [26, 28].

**Table 3:** Limit of blank, limit of detection and inter‐assay coefficient of variability for the targets.

Target	LOB (droplets)	LOB (meth %)	LOD (droplets)	LOD (meth %)	%CV
1. *EMX1*	2.8	0.31	7.2	0.84	4.3
2. Chr5q14.1	10.5	0.84	17.3	1.45	2.2
3. *NXPH1*	6.0	0.94	11.6	1.75	8.6

#### 2.5.1 Normalization and methylation level

The number of positive and negative droplets was normalized to 20 000, the theoretical number of droplets that can be obtained using the QX200™ system. As shown in Table S2, the number of accepted droplets was used for normalization.

The normalized number of droplets and the LOB were used to calculate the methylation percentage. This was calculated based on the number of droplets for the target sequences minus the LOB, and the reference sequence. After multiplying by 100, a percentage was obtained (see Table S2). This represents the relative abundance of the methylated target sequence. The ideal marker would show a methylation level of 100% for the positive control and 0% for the negative control. The methylation level can vary according to the number of droplets for the target sequence or reference sequence that are assigned in the clusters. In exceptional cases, the methylation level can exceed 100%, for example when copy number variants are present in a sample.

#### 2.5.2 Specificity and sensitivity

To calculate the specificity and sensitivity of the targets, first a receiver‐operator characteristic (ROC) model was constructed and then validated using five‐fold cross validation with the “ROCR” package in r (version 4.0.2, R Core team 2020, Vienna, Austria). The cross‐validated area under the ROC curve (cvAUC) was calculated to assess the predictive accuracy. Samples were considered methylated when the number of positive droplets for the target was higher than the LOB of that target. Samples were considered negative when (a) the number of positive droplets for the target was lower than the LOB and (b) no clear cluster for the target was observed. The sensitivities and specificities that were obtained per target and per cancer type are described in Table 4. For the combination of different targets, first the cut‐off per target was determined based on the highest overall accuracy, dichotomizing the results per target. Then, the separate predictions were combined by using the ‘prediction’ function of “ROCR”, merged ROC curves were constructed and cvAUC values were calculated. When combining several targets, sensitivities and specificities can be calculated in different ways. For the triplex assay, a sample was considered positive if one target out of the two was methylated, which will further be described as the ‘1/2 threshold’ (see also Fig. 4A). For the total ddPCR, which combines the three targets, two distinct thresholds were used. To achieve the highest sensitivity, a sample was considered positive based on methylation of one target out of three (further referred to as the ‘1/3 threshold’). For the highest specificity, a sample was only considered positive if the majority of the targets was methylated, i.e. two out of three (referred to as the ‘2/3 threshold’).

**Table 4:** Cross‐validated AUC and sensitivity‐specificity based on ROC analyses. 1/3 = sample is classified as tumor if there is a positive signal for one of the three targets, 2/3 = sample is classified as tumor if there is a positive signal for the majority of the targets (two out of three). cvAUC, cross‐validated area under the curve.

	Overall	Breast	Colorectal	Lung	Prostate	Pancreas	Liver	Esophagus	Head and neck
Target 1									
Sensitivity	82.5%	100%	28.6%	100%	100%	82.4%	66.7%	100%	53.8%
Specificity	98.2%	100%	100%	100%	91.7%	100%	100%	100%	100%
Target 2									
Sensitivity	76.6%	60%	100%	85%	78.6%	76.5%	66.7%	80%	69.2%
Specificity	93.6%	100%	70%	100%	91.7%	91.3%	100%	100%	100%
Triplex									
Sensitivity	93.2%	100%	100%	100%	100%	88.2%	83.3%	100%	76.9%
Specificity	92.7%	100%	70%	100%	87.5%	91.3%	100%	100%	100%
cvAUC	0.949	1	0.893	1	0.966	0.928	0.917	1	0.885
Target 3									
Sensitivity	74.3%	80%	100%	85%	57.1%	81.3%	41.7%	100%	61.5%
Specificity	91%	100%	40%	100%	91.7%	91.3%	100%	100%	100%
Total, 1/3									
Sensitivity	94.1%	100%	100%	100%	100%	87.5%	83.3%	100%	84.6%
Specificity	87.3%	100%	30%	100%	83.3%	87.0%	100%	100%	100%
Total, 2/3									
Sensitivity	80.2%	80%	100%	90%	85.7%	81.3%	58.3%	100%	53.8%
Specificity	96.4%	100%	80%	100%	95.8%	95.7%	100%	100%	100%
Total									
cvAUC	0.948	1	0.93	1	0.96	0.921	0.917	1	0.923

### 2.6 Statistical analysis

The graphpad prism (version 9.5.1, GraphPad Software LLC, Boston, MA, USA) software was used to perform statistical tests along with the generation of figures. To determine the sample size, we used the target with the smallest effect size. A sample size with seven cases and an equal number of controls holds 80% power to detect any difference between the tumor group (methylation level = 0.54 ± 0.23) and the normal group (methylation level = 0.18 ± 0.10), corrected for multiple testing (3 CpG sites) with a one‐sided test. More details are given in Table S2. To determine whether the difference that was observed between the methylation levels of DNA from normal adjacent samples and tumor samples was significant, the Mann–Whitney *U* test was used. To analyze whether there are differences in methylation levels between cancer stages, the Kruskal‐Wallis test was used. *Post‐hoc* analyses were performed using the Mann–Whitney *U* test.

### 2.7 Ethics approval and consent to participate

This study was conducted in accordance with Good Clinical Practice guidelines and the Declaration of Helsinki. Tissue samples were retrospectively collected from the Biobank of the Antwerp University Hospital for use in this study. Patients have given written informed consent for the use of their bodily material in research when they consented to the invasive procedure (according to article 20 of the Belgian Law on the procurement and use of human corporal material intended for human application or scientific research of 19 December 2008). All healthy volunteers have given written informed consent for the use of their bodily fluid in research. The UZA ethical committee approved the use of all samples (Ref. N°20/02/056 and Ref. N°41/14/426).

## 3 Results

### 3.1 Target selection and assay development

Previous bioinformatic analyses from TCGA by our group [8] demonstrated the use of differentially methylated CpG sites for discrimination between controls and different tumor types. From this study, several CpG targets were further investigated for their application as biomarkers. A total of three targets was retained. Primers and probes for the selected targets were first analyzed using qPCR (CFX384; Bio‐Rad) to determine their ability to discriminate hypermethylated samples (cancer cell lines) from hypomethylated samples (healthy blood samples). Two cell lines (HCT116 and Cal27) and two blood samples were analyzed in duplicate. The average *C*_q_ was used to determine the Δ*C*_q_. The qPCR results of these targets are displayed in Table S3. The minimal Δ*C*_q_ was 4.6, while the maximal Δ*C*_q_ was 11.3. Based on the three targets, two ddPCR assays were developed. ddPCR was the chosen method, because its detection sensitivity is crucial for the future application of the assays in liquid biopsies. Also, around 13% of samples have a low TcP (< 10%), for which we believe higher analytical sensitivity is beneficial. The ddPCR assays were optimized through temperature gradients and concentration gradients of probes. The first assay combines targets 1 and 2. For this assay, the optimal temperature was 55 °C. The optimal concentrations for probes were 450 nm for target 1, 680 nm for target 2 – FAM and 1.4 μm for target 2 – SUN in the final detection mix. The positive control (HCT116) had an LOB‐ corrected methylation percentage of 64.30% for target 1 and 71.62% for target 2 (Fig. 2A). The second assay consists of target 3. For this assay, the optimal temperature was 58 °C. The optimal concentration for the probe was 2.93 μm in the final detection mix. HCT116 has an LOB corrected methylation percentage of 73.79% (Fig. 2B).

### 3.2 Characteristics of the assays

For the different targets, several measures (LOB, LOD, and others) were calculated as described in Section 2.5.1 and Table S2. An overview of the parameters is given in Table 3. The %CV indicates great repeatability for targets 1 and 2 and good repeatability for target 3. In both assays, the average analytical detection sensitivity for the reference target was calculated over all samples (Table S2). For the triplex assay, the average detection sensitivity was 0.11 ± 0.04%. For the duplex assay, a very similar average detection sensitivity of 0.10 ± 0.05% was found, indicating an equally good performance for both assays. Finally, the number of accepted droplets and copies per droplet measured for the assays was evaluated. In the triplex assay, an average of 14 251 ± 1932 accepted droplets were found, with an average of 147 ± 86 albumin copies per droplet (Poisson‐corrected minimum) per run. For the duplex assay, the average of accepted droplets was 13 315 ± 1772 with on average 180 ± 121 albumin copies per droplets (Poisson‐corrected minimum) per run.

### 3.3 Methylation analysis of the targets in fresh frozen tumor and normal adjacent tissue

**Fig. 3:**
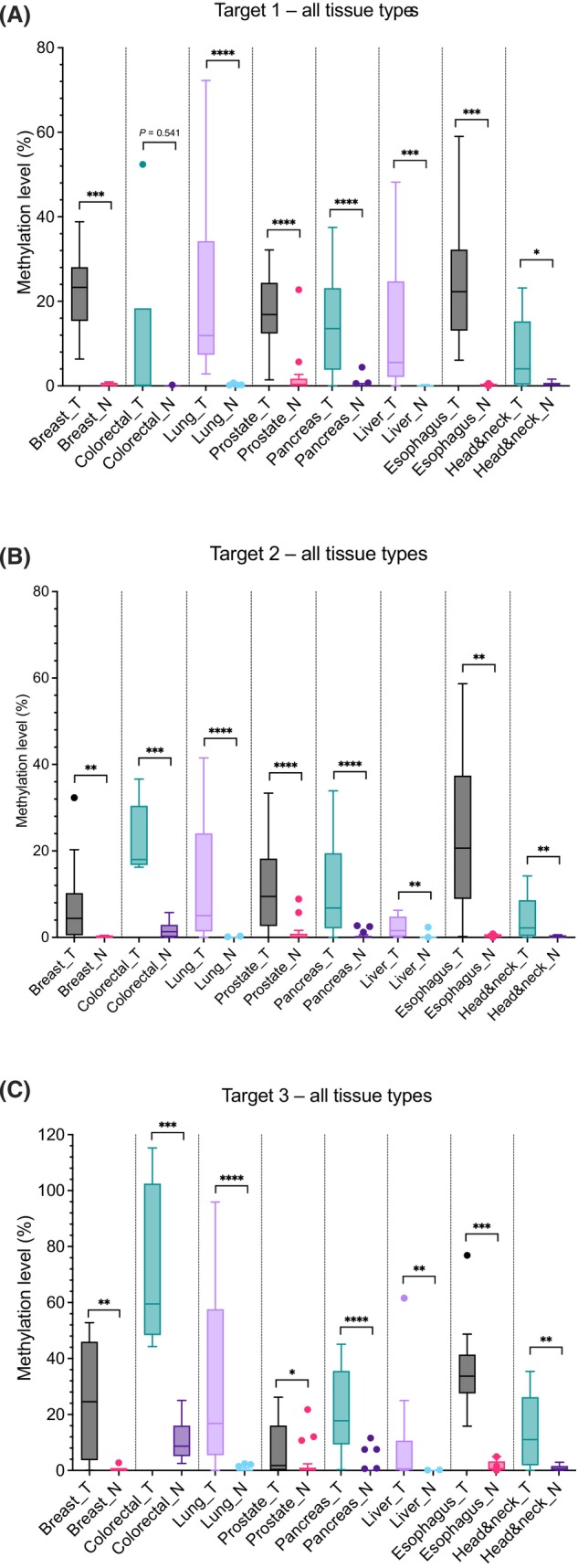
Overview of methylation levels in tumor and normal adjacent fresh frozen samples. (A) Overview for target 1. (B) Overview for target 2. (C) Overview for target 3. *P*‐values are indicated with asterisks, where *P < 0.05, **P < 0.01, ***P < 0.001 and ****P < 0.0001. N, normal adjacent; Tc, tumor. Mann–Whitney *U* tests were performed using graphpad prism. For the lines in the boxplots: error bars indicate the 95% confidence interval; The bottom and top of the box are the 25th and 75th percentiles, the line inside the box is the 50th percentile (median). Outliers are shown as closed circles.

The methylation levels of 103 tumor and 109 normal adjacent fresh frozen tissues were analyzed using both the triplex and the duplex assay. Methylation levels are summarized in Fig. 3. For target 1, all tissue types except colorectal tissue (*P* = 0.541) show a significant difference in methylation percentages between normal and tumor tissue. Lung, prostate and pancreatic tissue give the most significant results with *P*‐values below 0.0001. For target 2, all tissues have a significant difference in methylation levels. As for target 1, lung, prostate and pancreatic cancer give the most significant results again. For target 3, all tissues give significant differences in methylation levels for tumor samples compared to normal adjacent samples as well. Here, lung and pancreatic cancer have the most significant results, followed by colorectal and esophageal cancer.

Methylation levels in tumor tissue show a wide range from 0% to 72.2% for target 1, 0% to 58.7% for target 2 and 0% to 115% for target 3. In contrast, the normal adjacent tissue's range is limited from 0% to 5.7% for both targets 1 and 2 and 0% to 16.7% for target 3, excluding one outlier in the prostate group (22.7% for target 1, 8.9% for target 2 and 21.8% for target 3) and one in the colorectal group for target 3 (25%).

There is no strong correlation of the methylation levels between the different triplex targets for the same sample (*r* = 0.59 for tumor samples and *r* = 0.71 for normal samples, Fig. S2A,B). Furthermore, there is no correlation between the tumor cell percentage and the methylation levels of the tumor samples (*r* = 0.22 for target 1, *r* = 0.14 for target 2 and *r* = 0.12 for target 3, Fig. S2C–E). Last, there is no significant difference in methylation levels between different cancer stages for target 1 and target 3. For target 2, there is only a significant difference found in methylation levels between stages 1 and 3 (*P* = 0.03) (Fig. S2F–H). The diagnostic performance of the targets per cancer stage was evaluated by calculating the sensitivity from ROC analyses and is given in Table S4.

### 3.4 Sensitivity and specificity of the targets for multi‐cancer detection

**Fig. 4:**
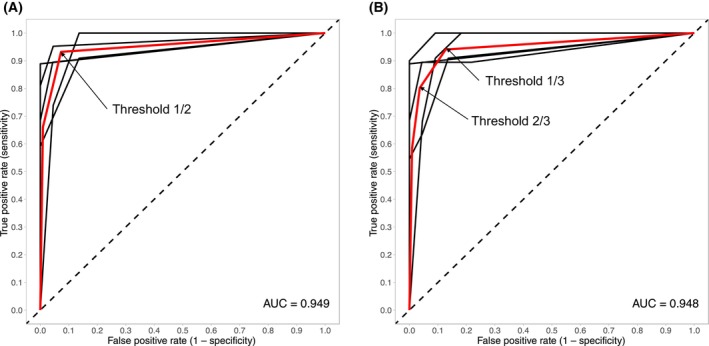
Receiver‐operator characteristic (ROC) curves. (A) ROC plot for the two targets of the triplex assay. (B) ROC plot combining all targets. AUC = (cross‐validated) area under the curve. For each target, the 5‐fold cross validation ROC curves (black) and the mean ROC curve (red) is plotted. Breakpoints reflect the number of targets agreeing in the classification. Depending on the numbers of targets (i.e. breakpoints) that classify tumor and normal samples correctly, the sensitivity and specificity varies. Arrows indicate the breakpoints (i.e. thresholds) that were used for calculations of the sensitivities and specificities.

To evaluate sensitivities and specificities for the targets, cross‐validated ROC plots were generated per tissue type, per target and for the combination of targets. Data from the separate targets is shown in Table 4 and ROC plots for the combination of the targets are shown in Fig. 4. They were used to determine the best threshold for sensitivity and specificity. For the combination of targets, samples were analyzed based on a 1/2, 1/3 and 2/3 threshold, as explained in the methods. A summary of the parameters can be found in Table 4.

In the multi‐cancer context, numbers indicate that, although targets perform well separately (see Table 4), combining targets can increase the sensitivity and specificity. For example, the overall sensitivity of target one is 82.5%, and for target 2, it is 76.6%. However, a sensitivity of 93.2% is reached when looking at both targets together. The cvAUC for the triplex is 0.949. The same is true for the overall sensitivity of target three, which is 74.3% but in the combination of all three targets, the overall sensitivity reaches 94.1% (1/3 threshold). The changes in specificity are less drastic, but also underline the strength of combining targets to improve the diagnostic value (see Table 4). Adding the third candidate‐biomarker target to the model, the total cvAUC reaches 0.948. Depending on the use of the 1/3 or 2/3 threshold, the model performs better for sensitivity or specificity respectively (see Table 4). Compared to the previously described *in silico* analyses by Ibrahim et al. [8], all three targets, the triplex and overall assay perform similarly (Table S5).

Looking at the cancer types separately, there are several cancer types for which the targets reach 100% sensitivity and 100% specificity. In contrast, some targets do not perform well in specific cancer types (see Table 4). However, as is also described above, combining at least two targets increases the sensitivity and specificity drastically (see Table 4).

## 4 Discussion

In this study, we successfully established two multi‐cancer detection ddPCR assays. Previous bioinformatic analyses by our group [8] have already demonstrated the use of differentially methylated CpG sites for discrimination between controls and multiple tumor types. From the analyses, multiple differentially methylated CpG sites were investigated. Selecting the sites based on the *P*‐value and discriminatory power (at least 20% difference in methylation level between tumor and normal samples) was an unbiased approach that left us with several candidate‐biomarkers. As ddPCR was the preferred method in view of future applications in liquid biopsies, several attempts in primer and probe design and testing have led to the ultimate retention of three targets.

Although the biomarkers used in our assays were selected purely on differential methylation, we investigated in which genes they are located. The first target covers a total of 17 differentially methylated CpGs in its amplified product. These sites are in the *EMX1* (Empty Spiracles‐Like Protein 1) gene [29]. Although this gene has no clear role in any carcinogenic processes, it has been reported to be differentially methylated in both gastric and hepatocellular carcinomas (HCC) [30, 31, 32, 33]. The second target covers six differentially methylated CpGs in total, in an intergenic region located on the short arm of chromosome 5 (Chr5q14.1). Loss of the chr5q arm has been described in myelodysplastic syndrome and several cancers [34, 35, 36, 37], but methylation at this specific location has not yet been described. In the third target region, a total of 9 differentially methylated CpGs were found. The gene at this location is the Neurexophilin 1 (*NXPH1*) gene. It is known to be involved in acute myeloid lymphoma [29]. Furthermore, it was described as a potential methylation biomarker in breast cancer [38] and intraductal papillary mucinous neoplasms (IPMNs) of the pancreas [39].

For the ddPCR analyses, two different types of assays were developed. In the triplex assay, target 1 has an LOB of 3, which is comparable with published ddPCR assays [20]. The %CV is low (4.3), indicating limited variability within the assay. Target 2 has a higher LOB and lower %CV (11 and 2.2 respectively). Targets 2 and 3 have a rather high LOD (methylation level of 1.45–1.75%), meaning that sufficient methylated DNA is needed for reliable detection of this target. Although all measures are within acceptable limits, the rather high LOB and LOD might hamper the application potential of this target in samples with very low tumor fractions (e.g. early stage liquid biopsies). Target 3 has an intermediate LOB of 6 and a %CV of 8.6, which again indicates good agreement within the assay. All targets have a standard error of the mean of approximately 1.5, indicating good repeatability. Last, the analytical detection sensitivity of the triplex assay and duplex assay is very similar (0.10% and 0.11%), indicating that both assays perform equally well. In the analyses, clusters were assigned manually for both assays. This remains standard practice for multiplex assays and the characterization parameters did not implicate this is as a problem. The manual assignment of the clusters can slightly affect the absolute quantification. However, for this assays' purpose, the relative quantification (i.e. methylation level) is of importance. Using manual assignment of clusters to have positive droplets only in the designated cluster, allowed us to minimize the LOB of the targets and as such minimize the number of false positive droplets. This is more important for the clinical implementation than the absolute quantification, in this specific assay context. Nevertheless, manual assignment could make standardization among different laboratories more difficult, and standardized methylation analyses are necessary for implementation in the clinic. In this view, Jeanmougin et al. [40] described PoDcall, an R package for automated calling of positive droplets, quantification and normalization of methylation levels. However, the paper was only published in 2022, so it might need more testing and validation before it can be implemented in other studies as a standard tool. Furthermore, in these analyses, we only verify positive clusters. This might lead to a minor decrease in sensitivity, which might be unfavorable for cfDNA analysis. However, Whale et al. [25] previously described that unbiased estimates can be achieved by using either all partitions or a subset of partitions, as long as no linkage between the targets occurs. Since our targets are located on different chromosomes, they are not linked and we can expect unbiased results by using only positive clusters.

The optimized triplex and duplex ddPCR assays were used to assess fresh frozen tissue samples. For all tissue types and targets, except target 1 in colorectal tissue, a significant difference between tumor and normal adjacent samples was found. Lung tissue and pancreatic tissue had the most significant results in all targets. As the tumor cell percentages and cancer stages were in the same range for all tissue types, this is most likely due to their larger sample size (*n* = ~ 20) compared to other tissue types (*n* = ~ 10). In most of the normal tissue groups, the methylation levels are not 0% but vary. Normal adjacent tissue was used, which was taken at a certain (unknown) distance from the tumor. Field cancerization could be a possible explanation for higher methylation levels in normal adjacent tissue [41]. This is most likely for target 3 in colorectal normal tissue, where methylation levels vary from 2.5% to 25%. Nonetheless, for this target, the colorectal tumor samples also show high methylation levels, making it possible to perfectly distinguish between tumor and normal samples. Head and neck tissue remains the most difficult tissue type with the lowest sensitivity (see Table 4) compared to other tissue types. DNA methylation is tissue type specific, and head and neck cancers comprise several tissues (mouth, sinuses, nose and throat). Importantly, the etiology of the different sub‐groups in head and neck cancer varies. These reasons could possibly explain why the targets are not as sensitive in head and neck cancers as in other tissue types. Finally, for esophagus cancer the number of normal adjacent tissues (*n* = 5) does not meet the criteria from the sample size calculation (*n* = at least 7). Further analyses with more samples are warranted to draw robust conclusions for this subgroup.

Although we expected to find a correlation between the estimated TcP and the methylation level per target, there was none. As outlined in the material and methods section, HE‐slides were reviewed by a pathologist (D.P.) to verify the tissue type and estimate the percentage of tumor cells. Since this material is precious, only one slide was used for verification. Unfortunately, estimating the tumor cell percentage stays a bit arbitrary and subjective. Moreover, the presence of other cells within the tumor samples, such as normal cells and immune cells (originating from inflammatory processes), dilute the number of tumor cells which affects the estimated TcP to an uncertain extent. Lastly, this correlation was made between the methylation level of one location in the epigenome and the overall TcP of the tumor sample, which might hamper the potential to find a correlation.

In literature, methylation specific ddPCR assays typically involve the detection of one target gene in one cancer type. Almost all papers describe the use of ddPCR assays in both tissue and liquid biopsies (cfDNA) [6, 7, 14, 17]. In more recent research, the combination of several targets in separate ddPCR duplex assays is often described [17]. However, by creating separate assays, more DNA input is needed compared to our triplex assay. Our triplex assay could be beneficial for low DNA input samples or samples with limited tumor fractions such as cfDNA, as two targets can be investigated using the same amount of DNA. Compared to qPCR, ddPCR has a better analytical detection sensitivity (1% vs 0.001%) which is important for cfDNA or FFPE samples and early detection. As observed in our experiments (see Table S6), the LODs of ddPCR were far better than those of the qPCR (~ 1% vs ~ 6% respectively). In view of future clinical applications, which can be both FFPE and liquid biopsy related, we designed ddPCR assays and optimized them using fresh frozen material. The advantage is that both ddPCR assays are now readily available and validated.

Furthermore, combining targets in one assay increases the sensitivity as can be appreciated by comparing data from Table 4 (targets 1 and 2 vs triplex). Table 4 shows that not all targets perform equally well in all cancer types. However, we use this as a strength of our overall assay. By combining all three targets from two ddPCR assays, we were able to achieve high sensitivities and specificities for all cancer types. To allow for different applications, both the 1/3 and 2/3 thresholds were used to calculate diagnostic parameters. As seen in Table 4, the sensitivity is higher when using the 1/3 threshold approach. In contrast, the 2/3 threshold approach allows for higher specificity.

The use of multiplex ddPCR assays in cancer has been described for microsatellite instability, mutation, and copy number alteration detection [42, 43, 44, 45, 46, 47]. Methylation detection has been used more in other fields such as detection of fetal fraction in blood samples and detection of SARS‐CoV‐2 [48, 49]. There is one cancer multiplex methylation ddPCR assay described in literature, which was used to detect differences in methylation before and after neoadjuvant therapy in breast cancer [50]. Here, they used different concentrations of the FAM probe for the detection of two target genes in one assay. In addition, there is only one more assay described very recently by Zhao et al. [51] for lung cancer screening. Despite their capability of combining four targets into one assay, the use of their own microfluidics device limits broad applications in the clinic.

Regarding multi‐cancer applications, we are the first to describe a multi‐cancer ddPCR assay using multiplexing. There is one recent paper by Manoochehri et al. [52] that describes the use of the *SST* gene as an interesting marker for several cancer types, including all types described in our study. However, only pancreatic cancer tissue was evaluated *in vitro* with a ddPCR assay. The AUC for this was 1. The combination of our three targets reached an AUC of 0.957 for pancreatic tissue and is thus comparable to the *SST* biomarker performance. The other cancer types have only been described in an *in silico* test for which no AUCs were calculated, so we cannot compare the *SST* gene to our targets for multi‐cancer detection [52]. Other multi‐cancer detection assays described in literature, e.g. the Galleri test from GRAIL, are NGS based [21]. However, in view of implementation in the clinic, ddPCR is less labor intensive and more cost‐efficient than NGS [18]. Multi‐cancer assays often also incorporate the detection of tissue of origin (TOO), which our assay does not specify.

## 5 Conclusions

In conclusion, we are the first to report a multi‐cancer multiplex methylation ddPCR assay. The overall assay with three methylation biomarkers reached an AUC of 0.948 in eight different tumor types. In the future, more multiplexing is likely to be achieved with the novel QX600 system of Bio‐Rad, where 6 fluorescent channels will allow multiplexing of approximately 10 targets, which could greatly enhance sensitivity. Furthermore, the assays need to be evaluated in FFPE material and liquid biopsies to assess their performance in (poor quality) DNA from FFPE samples and cfDNA, as this will be of more interest to be implemented in the clinic. The results of this study can serve as a solid basis for further MCED‐ddPCR cfDNA applications.

## Conflict of interest

The authors declare no conflict of interest.

## Author contributions

The work reported in the paper has been performed by the authors, unless clearly specified in the text. IN contributed to conceptualization of the study, methodology, interpreting results, writing original draft, reviewing and editing of the final draft. NDM contributed to methodology, interpreting results, reviewing and editing of the final draft. JV, TVP, and GVC contributed to conceptualization of the study, interpreting results, reviewing and editing of the final draft. DP contributed to methodology (histopathological analysis), reviewing and editing of the final draft. MP contributed to conceptualization of the study, reviewing and editing the final draft. KOB contributed to conceptualization of the study, interpreting results, reviewing and editing the final draft. All authors have read and agreed to the published version of the manuscript.

### Peer review

The peer review history for this article is available at https://www.webofscience.com/api/gateway/wos/peer‐review/10.1002/1878‐0261.13708.

## Supporting information


**Fig. S1.** Dispersion graphs of negative samples in the triplex and duplex assay.
**Fig. S2.** Correlations of methylation levels per target.
**Fig. S3.** Different probe concentrations for cluster separation.
**Table S1.** Amplification protocol.
**Table S2.** Calculations.
**Table S3.** Overview of qPCR results.
**Table S4.** Sensitivity of targets per cancer stage (ROC analysis).
**Table S5.** Comparison of the targets to *in silico* analyses of Ibrahim et al.
**Table S6.** Information regarding LOD‐LOB of qPCR.

## Data Availability

All data generated or analyzed during the study are included in this published article and its supplemental information files.
