# Supplementary material for: Simultaneous detection of eight cancer types using a multiplex droplet digital PCR assay
Source: Mol Oncol. 2024 Sep 6;19(1):188–203. doi: 10.1002/1878-0261.13708 (PMC11705734; doi:10.1002/1878-0261.13708)
Supplement: Supplementary file 1 — Fig. S1. Dispersion graphs of negative samples in the triplex and duplex assay. Fig. S2. Correlations of methylation levels per target. Fig. S3. Different probe concentrations for cluster separation. Table S1. Amplification protocol. Table S2. Calculations. Table S3. Overview of qPCR results. Table S4. Sensitivity of targets per cancer stage (ROC analysis). Table S5. Comparison of the targets to in silico analyses of Ibrahim et al. Table S6. Information regarding LOD‐LOB of qPCR. [file MOL2-19-188-s001.zip › mol213708-sup-0005-TableS2.pdf]

Suppl Table 2: Calculations made using the output file of the QuantaSoft™ software.

Sample Size Calculation*: Effect Size	$d = \frac{(M2 - M1)}{\sqrt{\frac{(SD1^2 + SD2^2}{2}}}$
Normalization of the number of droplets	$\frac{\text{Number of positive or negative droplets}}{\text{Number of accepted droplets}} * 20\,000$
Calculation of the methylation percentage	$\frac{\text{Normalized number of droplets target sequence}}{\text{Normalized number of droplets reference sequence}} * 100$
Limit of blank (LOB)	$\text{Mean number positive droplets}_{\text{blank}} + 1.645\ (\text{SD}_{\text{blank}})$
Limit of Detection (LoD)	$\text{LOB} + 1.645\ (\text{SD}_{\text{low sample concentration}})$
Calculation of the normalized number of droplets corrected with the LOB	$\frac{(\text{Number of positive or negative droplets} - \text{LOB})}{\text{Number of accepted droplets}} * 20\,000$
Calculation of the methylation percentage corrected with the LOB	$\frac{\text{LOB corrected number of normalized droplets target sequence}}{\text{Normalized number of droplets reference sequence}} * 100$
Inter-assay variability coefficient (%CV)	$\frac{\text{Average } \frac{ng}{\mu L} \text{ of triplicates}}{\text{Standard deviation } \frac{\text{conc}}{\mu\text{L}} \text{ of triplicates}} * 100$
Detection sensitivity (%)	$\frac{\textit{Haploid genome equivalents } (3)}{\textit{Number of copies per well}} * 100$

* The sample size calculation is performed as described in Rosner B. Fundamentals of Biostatistics. 7th ed. Boston, MA: Brooks/Cole; 2011. For this, we need power, effect size and significance level. The power was set to 80%. The formula for the effect size is given in this table (M= mean, SD= standard deviation, 1= tumor group, 2= normal adjacent group). We chose the target with the smallest effect size (target 3, with an effect size of 2.21 in comparison to target 1 (2.31) and target 2 (2.8)). The significance level was 0.05. As there are three targets, we corrected for multiple testing using a one-sided test, since the targets are chosen to be hypermethylated in tumor, so we already know one group will have higher methylation levels.
