# Supplementary material for: Simultaneous detection of eight cancer types using a multiplex droplet digital PCR assay
Source: Mol Oncol. 2024 Sep 6;19(1):188–203. doi: 10.1002/1878-0261.13708 (PMC11705734; doi:10.1002/1878-0261.13708)
Supplement: Supplementary file 1 — Fig. S1. Dispersion graphs of negative samples in the triplex and duplex assay. Fig. S2. Correlations of methylation levels per target. Fig. S3. Different probe concentrations for cluster separation. Table S1. Amplification protocol. Table S2. Calculations. Table S3. Overview of qPCR results. Table S4. Sensitivity of targets per cancer stage (ROC analysis). Table S5. Comparison of the targets to in silico analyses of Ibrahim et al. Table S6. Information regarding LOD‐LOB of qPCR. [file MOL2-19-188-s001.zip › mol213708-sup-0009-TableS6.docx]

**Supplementary Table 6 – LOB and LOD of the qPCR**

| **Target** | **LOB (%)** | **LOD (%)** |
| --- | --- | --- |
| *EMX1* | 5.46 | 6.84 |
| Chr5q14.1 | 4.36 | 7.31 |
| *NXPH1* | 3.28 | 5.30 |

To determine the LOB and LOD for qPCR, a DNA titration experiment using human methylated and non-methylated (WGA) DNA (Zymo Research, CA, USA) was performed. Three replicates of concentrations between 0% and 5% were measured with the assays to determine the LOB and LODs. They were calculated as described in literature (see also supplementary Table 2). To determine the corresponding methylation level, linear regression was used. The LOBs and LODs are given below.
