# Supplementary material for: Simultaneous detection of eight cancer types using a multiplex droplet digital PCR assay
Source: Mol Oncol. 2024 Sep 6;19(1):188–203. doi: 10.1002/1878-0261.13708 (PMC11705734; doi:10.1002/1878-0261.13708)
Supplement: Supplementary file 1 — Fig. S1. Dispersion graphs of negative samples in the triplex and duplex assay. Fig. S2. Correlations of methylation levels per target. Fig. S3. Different probe concentrations for cluster separation. Table S1. Amplification protocol. Table S2. Calculations. Table S3. Overview of qPCR results. Table S4. Sensitivity of targets per cancer stage (ROC analysis). Table S5. Comparison of the targets to in silico analyses of Ibrahim et al. Table S6. Information regarding LOD‐LOB of qPCR. [file MOL2-19-188-s001.zip › Supplementary information.docx]

**Supplementary information**

**Supplementary information**

**Supplementary Tables**

**Supplemental Table 1: Amplification protocol.** This file contains the full droplet digital PCR cycling protocol

**Supplemental Table 2: Calculations**. This file contains an overview of all the calculations made using the output file of the QuantaSoftTM software.

**Supplemental Table 3:** **Overview of qPCR results**. This file contains the results obtained for all targets with qPCR.

**Supplemental Table 4: Sensitivity of targets per cancer stage (ROC analysis)**

**Supplementary Table 5: Comparison of the targets to *in silico* analyses of Ibrahim *et al.***

**Supplementary Table 6: Information regarding LOD-LOB of qPCR**

**Supplementary Figures**

**Supplemental figure 1: Dispersion graphs of negative samples in the triplex and duplex assay.** The graphs show the dispersion of droplets for a negative control (gDNA from a whole blood sample) A) Triplex assay consisting of target 1, 2 and reference Albumin. B) Duplex assay consisting of target 3 and reference albumin.

**Supplemental figure 2: Correlations of methylation levels per target.** A-B) Methylation levels in the triplex assay. C-E) Methylation levels of samples and tumor cell percentage (TcP). F-H) Methylation levels per cancer stage. Kruskal-Wallis tests and post-hoc Mann-Whitney U tests were used. Ns = not significant, *= p-value < 0.05. Analyses and plotting were performed using GraphPad Prism.

**Supplemental figure 3: Different probe concentrations for cluster separation.** This figure illustrates the increased cluster separation at different probe concentrations for the triplex and the duplex assay. Probe concentrations are given in the figure.
