# Supplementary figures and images for: Simultaneous detection of eight cancer types using a multiplex droplet digital PCR assay
Source: Mol Oncol. 2024 Sep 6;19(1):188–203. doi: 10.1002/1878-0261.13708 (PMC11705734; doi:10.1002/1878-0261.13708)

**(A)**

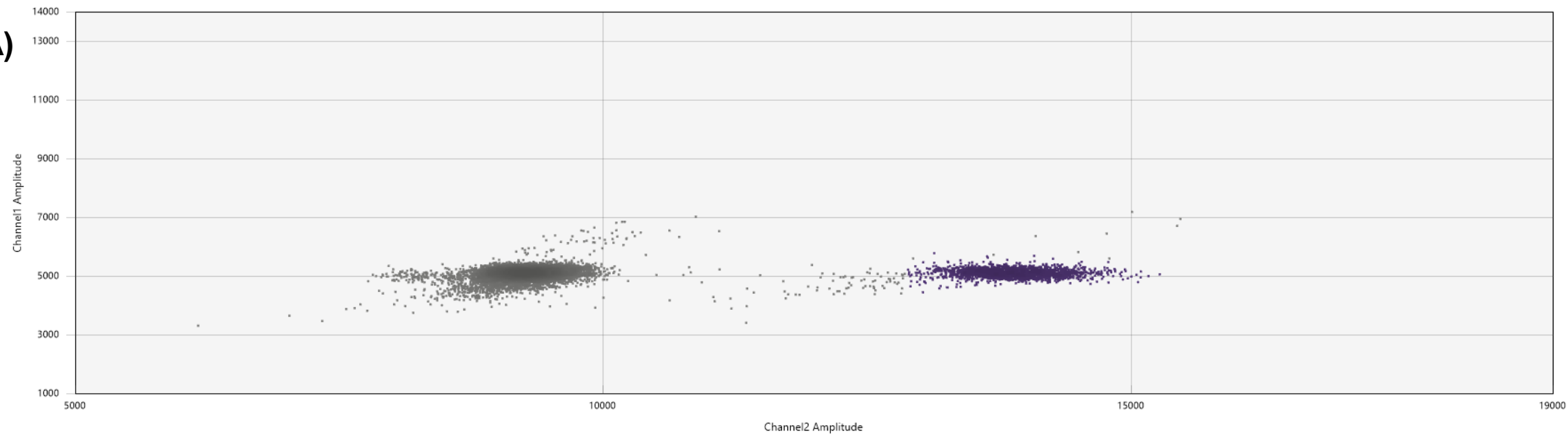

**(B)**

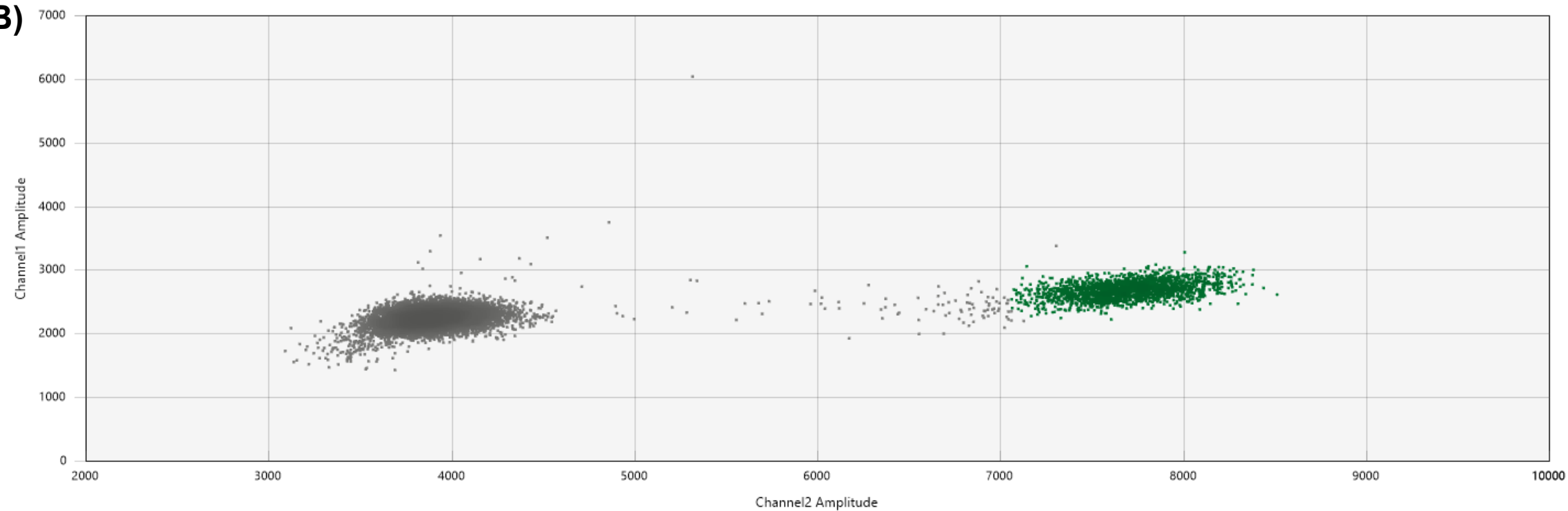

Supplement: Supplementary file 1 — Fig. S1. Dispersion graphs of negative samples in the triplex and duplex assay. Fig. S2. Correlations of methylation levels per target. Fig. S3. Different probe concentrations for cluster separation. Table S1. Amplification protocol. Table S2. Calculations. Table S3. Overview of qPCR results. Table S4. Sensitivity of targets per cancer stage (ROC analysis). Table S5. Comparison of the targets to in silico analyses of Ibrahim et al. Table S6. Information regarding LOD‐LOB of qPCR. [file MOL2-19-188-s001.zip › mol213708-sup-0001-FigS1.pdf]

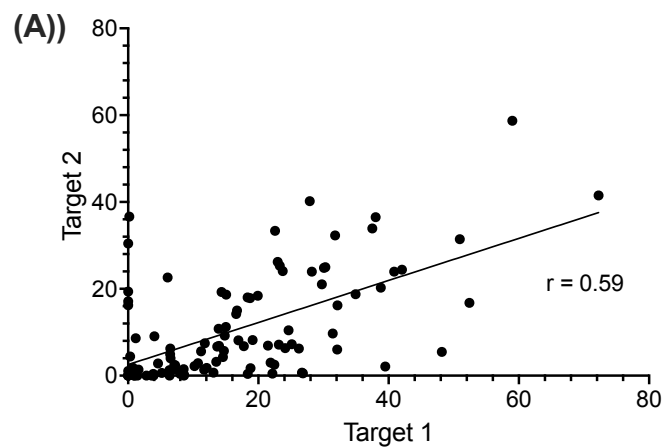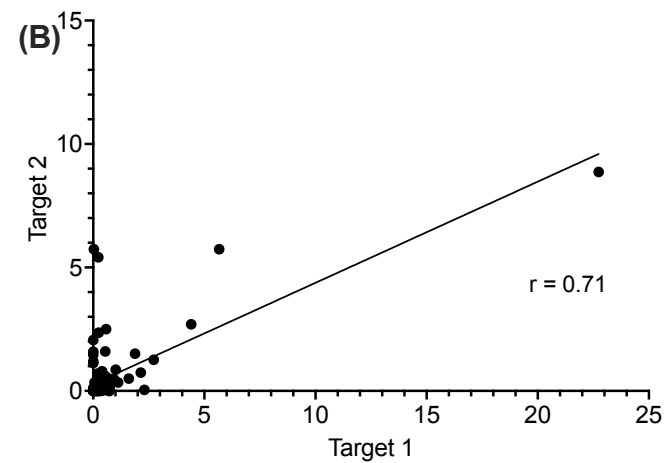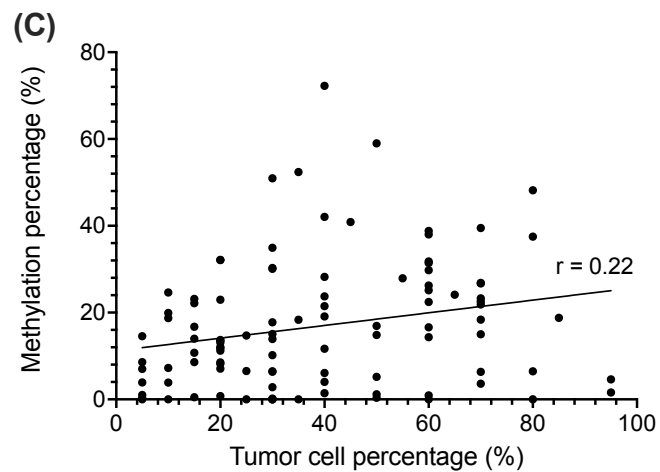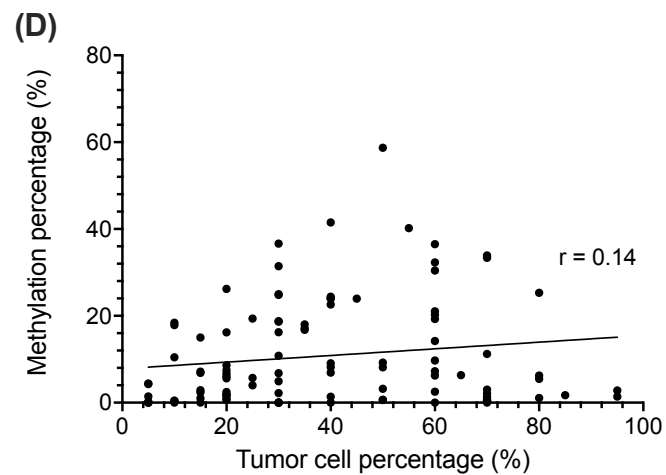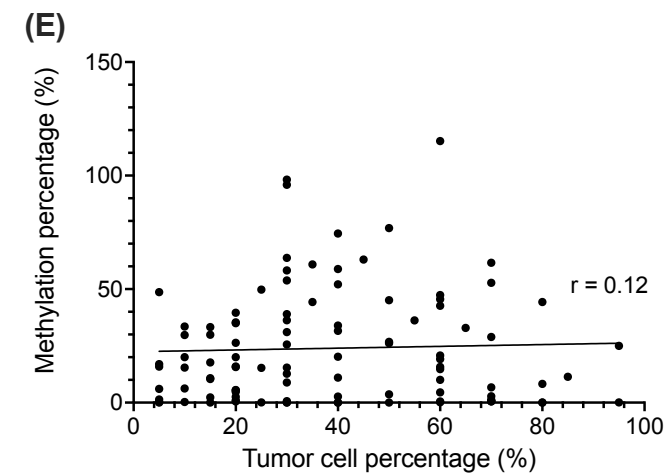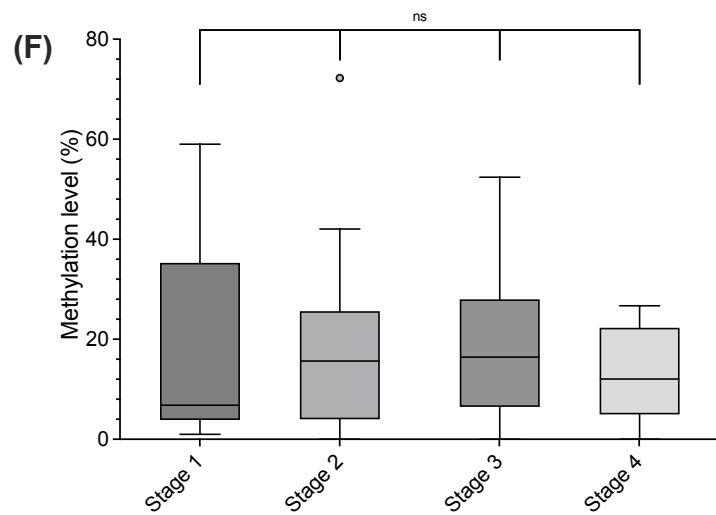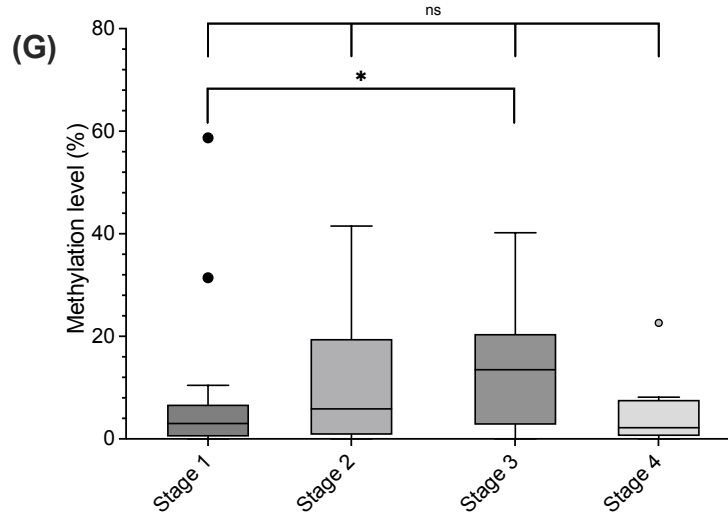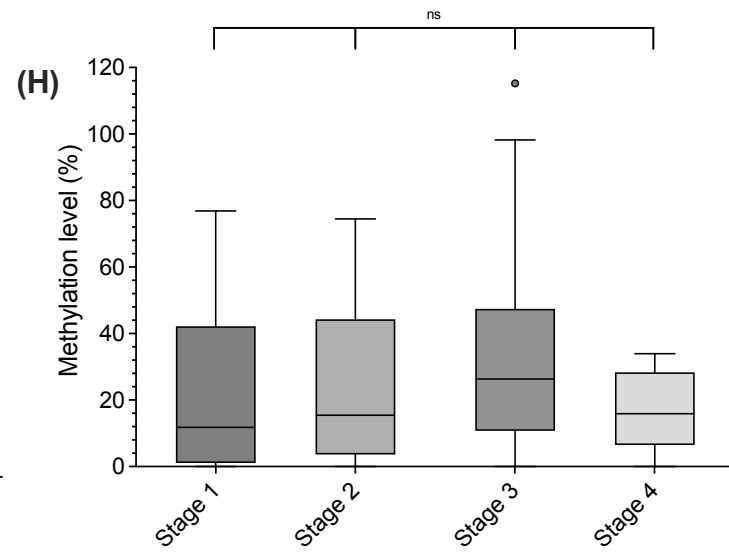

Supplement: Supplementary file 1 — Fig. S1. Dispersion graphs of negative samples in the triplex and duplex assay. Fig. S2. Correlations of methylation levels per target. Fig. S3. Different probe concentrations for cluster separation. Table S1. Amplification protocol. Table S2. Calculations. Table S3. Overview of qPCR results. Table S4. Sensitivity of targets per cancer stage (ROC analysis). Table S5. Comparison of the targets to in silico analyses of Ibrahim et al. Table S6. Information regarding LOD‐LOB of qPCR. [file MOL2-19-188-s001.zip › mol213708-sup-0002-FigS2.pdf]

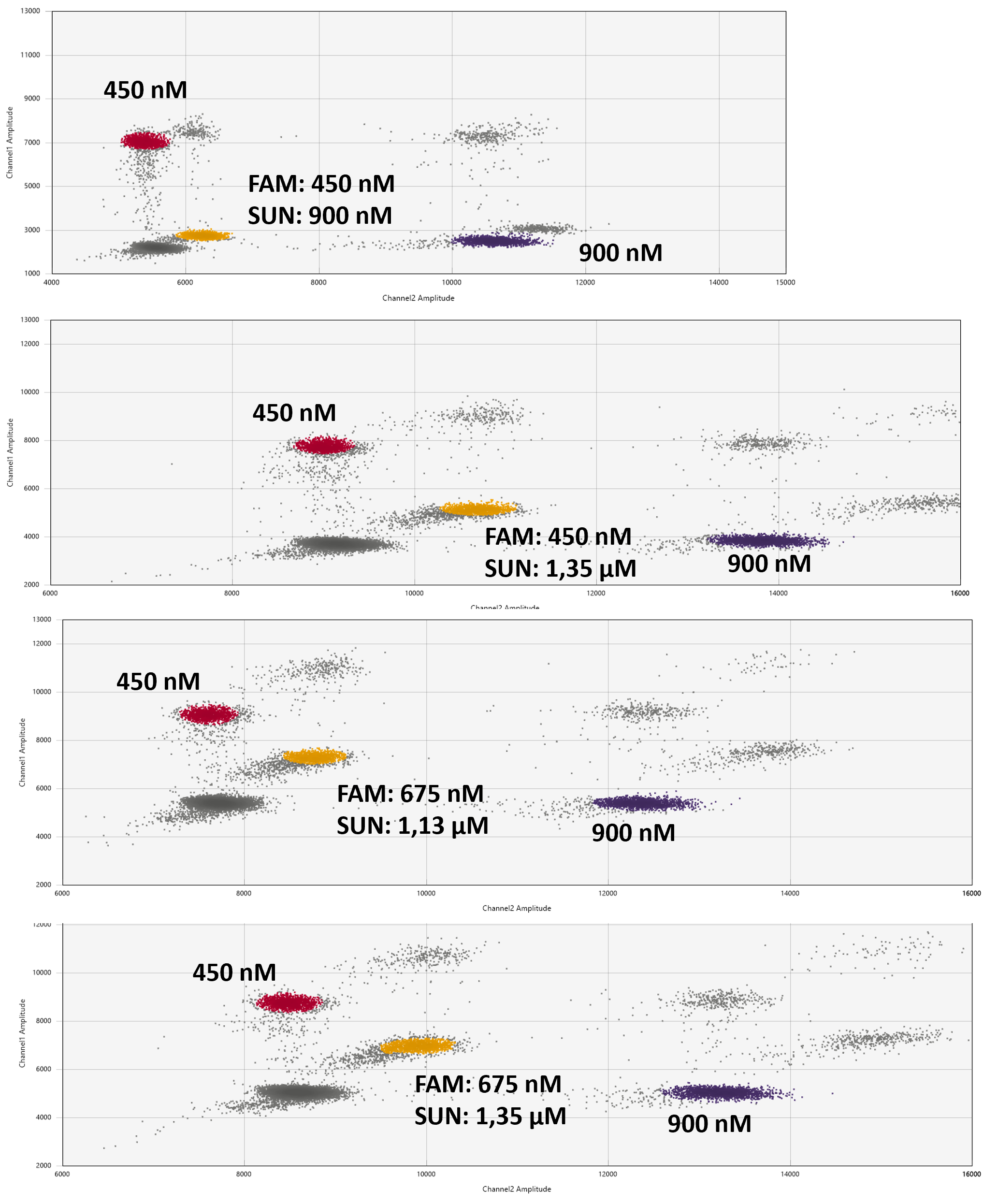

Supplement: Supplementary file 1 — Fig. S1. Dispersion graphs of negative samples in the triplex and duplex assay. Fig. S2. Correlations of methylation levels per target. Fig. S3. Different probe concentrations for cluster separation. Table S1. Amplification protocol. Table S2. Calculations. Table S3. Overview of qPCR results. Table S4. Sensitivity of targets per cancer stage (ROC analysis). Table S5. Comparison of the targets to in silico analyses of Ibrahim et al. Table S6. Information regarding LOD‐LOB of qPCR. [file MOL2-19-188-s001.zip › mol213708-sup-0003-FigS3.png]
